# Rapunzel Syndrome in a Teenage Girl: A Case Report

**DOI:** 10.7759/cureus.29975

**Published:** 2022-10-06

**Authors:** Barath Prashanth Sivasubramanian, Mahalakshmi Ashokkumar, Mohamed Afzal, Vikramaditya Samala Venkata, Uma D Dhanasekaran, Sandhya R Palit, Viraj Panchal, Diviya Bharathi Ravikumar, Srikanth Puli, Vanitha Krishnaswamy

**Affiliations:** 1 Department of Internal Medicine, Employees' State Insurance Corporation Medical College and Post Graduate Institute of Medical Science and Research, Chennai, IND; 2 Department of General Surgery, KAP Viswanathan Government Medical College, Trichy, IND; 3 Department of Internal Medicine, Dartmouth Hitchcock Cheshire Medical Center, Keene, USA; 4 Department of General and Colorectal Surgery, Employees' State Insurance Corporation Medical College and Post Graduate Institute of Medical Science and Research, Chennai, IND; 5 Department of Internal Medicine, Smt. Nathiba Hargovandas Lakhmichand Municipal Medical College, Ahmedabad, IND; 6 Hospital Medicine, Dartmouth Hitchcock Cheshire Medical Center, Keene, USA; 7 Department of Pediatrics, KAP Viswanathan Government Medical College, Trichy, IND

**Keywords:** abdominal pain, trichobezoar, trichophagia, obsessive-compulsive disorder, rapunzel syndrome, trichotillomania

## Abstract

Trichobezoars are concretions of retained, undigested material, mostly hair or hair-like fibers in the stomach. Rapunzel syndrome is the condition when trichobezoars extend into the small bowel, leading to various complications including bowel obstruction, and perforation due to pressure necrosis.

We present an interesting case of trichobezoar in a 15-year-old female patient, who presented with abdominal pain for one month duration. The exam was notable for a firm palpable mass in the epigastric area associated with localized tenderness and guarding. Contrast-enhanced CT of the abdomen showed a distended stomach with the bezoar, dilated duodenal loops, and clumping of proximal jejunal loops. Upper gastrointestinal endoscopy showed a trichobezoar extending from the oesophagogastric junction to the pylorus. Endoscopic removal of the trichobezoar was not successful.

An elective laparotomy was performed, during which the stomach, duodenum, and proximal jejunum were dilated. The trichobezoar, measuring 35 cm in length, extended from the body of the stomach to the proximal jejunum and caused jejunal perforation due to pressure necrosis. The trichobezoar was removed and primary closure of jejunal perforation with diversion gastrojejunostomy and jejunostomy was done. There were no postoperative complications. The patient followed up with psychiatry in the clinic after discharge, she was diagnosed with trichotillomania and started on fluoxetine, with improvement in her behavioral symptoms.

In young female patients with nonspecific chronic abdomen pain and a palpable mass, trichobezoar should be considered in the differential diagnosis. Contrast-enhanced abdomen CT is the preferred imaging modality and removal of the trichobezoar with an appropriate endoscopic or surgical procedure is the treatment of choice. It is essential to diagnose and treat the underlying behavioral condition to prevent recurrent episodes.

## Introduction

Bezoars are defined as concretions of retained, undigested material found in the stomach, the ingested material in the stomach may include plant fibers (phytobezoars), persimmon (diospyrobezoar), hair (trichobezoar), milk protein (lactobezoar), paper, medications (pharmacobezoar) [1]. Trichobezoars are bezoars formed due to the ingestion of hair or hair-like fibers [2]. Trichobezoars are usually found in the stomach, but as they enlarge over time, they can extend into the small bowel. When trichobezoars reach the small bowel, the condition is then called Rapunzel syndrome [3]. Trichotillomania is a psychiatric disorder characterized by a constellation of symptoms including obsessive thoughts, compulsive behaviors like repeated hair plucking, hair ingestion, etc and is associated with Rapunzel syndrome [4-8]. According to the reports, trichotillomania affects up to 4% of the population, with the highest incidence found in children and adolescents [9]. Among patients with trichotillomania, 87% of individuals have obsessive-compulsive disorder, 64% have mood disorders, 52% have a generalized anxiety disorder, and 44% have skin-picking disorder as a comorbid condition [10]. 

It is quite rare to have an intestinal blockage during childhood from bezoars, the most frequently found materials are hairs (trichobezoar) and alimentary fibers (phytobezoar) [11]. Rapunzel Syndrome is a rare form of trichobezoar in which the intestinal blockage is caused by the swallowed hair fibers, extending like a tail from the stomach into the small bowel [12]. Trichobezoar was first reported in the 18th century and since then, there have only been a few occurrences documented in the literature [3,4,10,12-16]. Early on, trichobezoars might cause symptoms that are not specific [13]. Bezoars have rarely been linked to gastrointestinal complications such as intestinal perforation, peritonitis, protein-losing enteropathy, steatorrhea, pancreatitis, intussusception, obstructive jaundice, appendicitis, constipation, and pneumatosis intestinalis [17,18]. Here, we present a case of Rapunzel syndrome with jejunal perforation, found in a 15-year-old female patient with no significant past medical history.

## Case presentation

**Figure 1:**
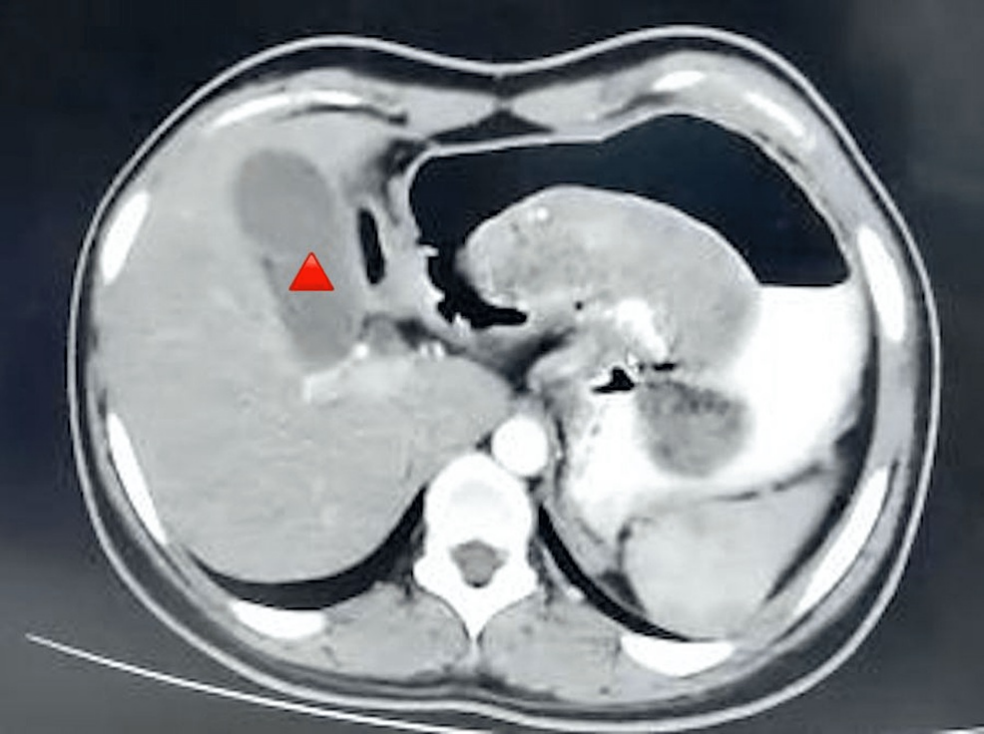
Distension of Stomach With Trichobezoar, CT image Contrast-enhanced CT of the abdomen showed a distended stomach with the bezoar

A 15-year-old girl presented with dull abdominal pain for one month, accompanied by multiple episodes of emesis for one week, with no prior comorbid conditions. On physical examination, the patient appeared well nourished, vital signs showed a heart rate of 94 beats/minute, respiratory rate of 15 breaths/ minute, and blood pressure of 110/70 mm Hg. On local examination, the abdomen was tender in the epigastric and umbilical regions. A firm mass was palpable in the epigastric area along with localized guarding. Bowel sounds were audible. Per rectal examination was normal. Lab results revealed a stable complete blood count, basic metabolic profile, liver function test values, normal bleeding time, and a normal coagulation profile. Contrast-enhanced CT of the abdomen showed a distended stomach with the bezoar (Figure 1).

**Figure 2:**
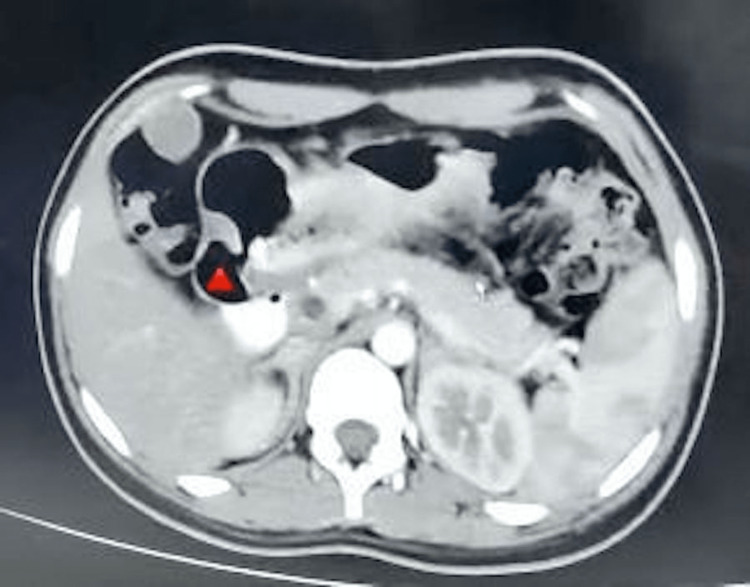
Dilated Bowel Loops, CT image Dilated bowel loops with clumping of bowel loops involving proximal jejunal loops with normal distal flow contrast into distal loops

Dilated duodenal loops with clumping of bowel loops were noted in the left hypochondrium involving proximal jejunal loops with normal distal flow contrast into distal loops (Figure 2).

**Figure 3:**
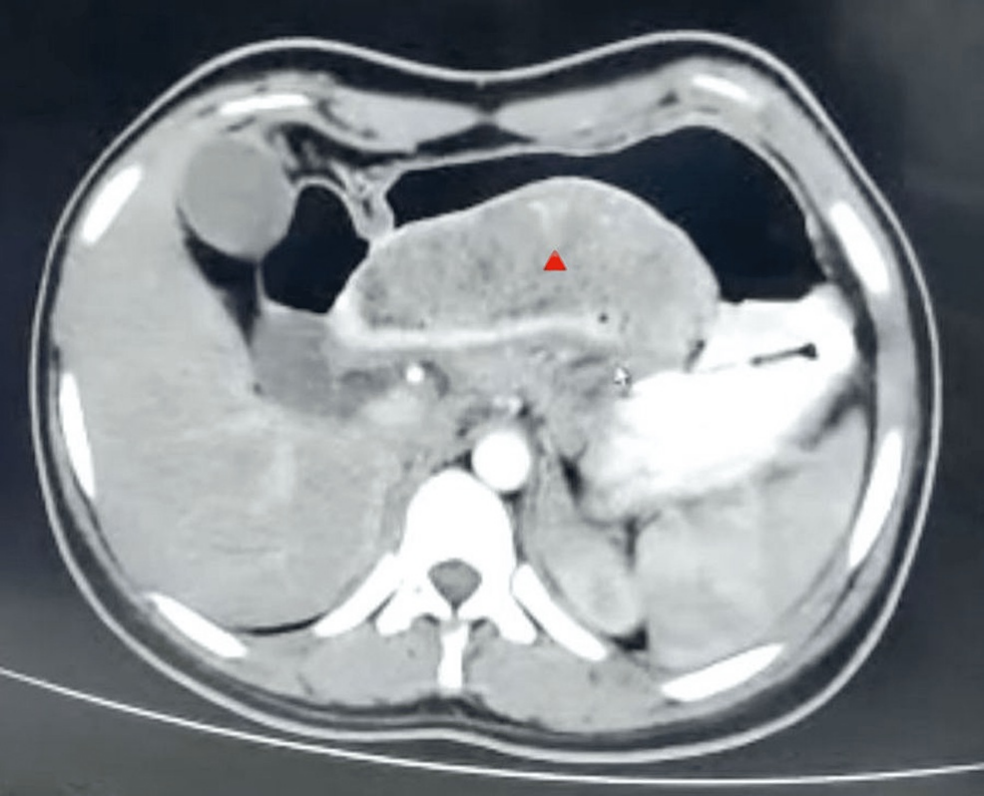
Trichobezoar in Duodenum, CT image Contrast-enhanced CT showed bezoar in the duodenum and dilated intestinal loops

**Figure 4:**
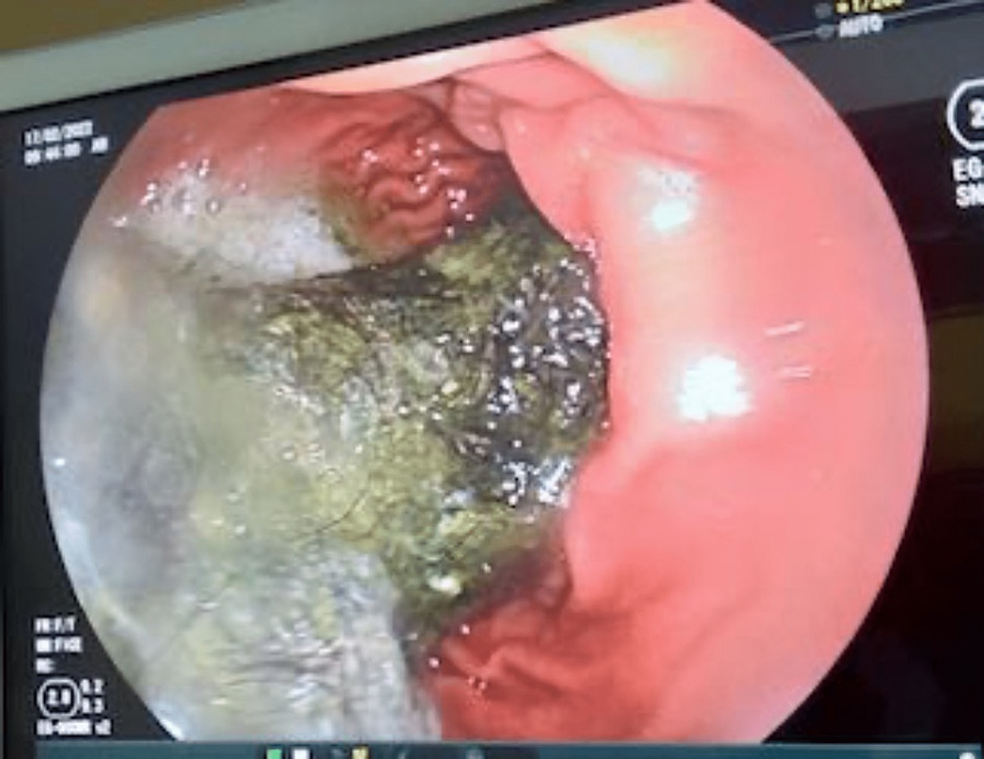
Endoscopy On upper gastrointestinal endoscopy, bezoar was found just below the oesophagogastric junction, extending through the body of the stomach, its antrum, and pylorus

On upper gastrointestinal endoscopy, bezoar was found just below the oesophagogastric junction, extending through the body of the stomach, its antrum, and pylorus (Figure 4).

**Figure 5:**
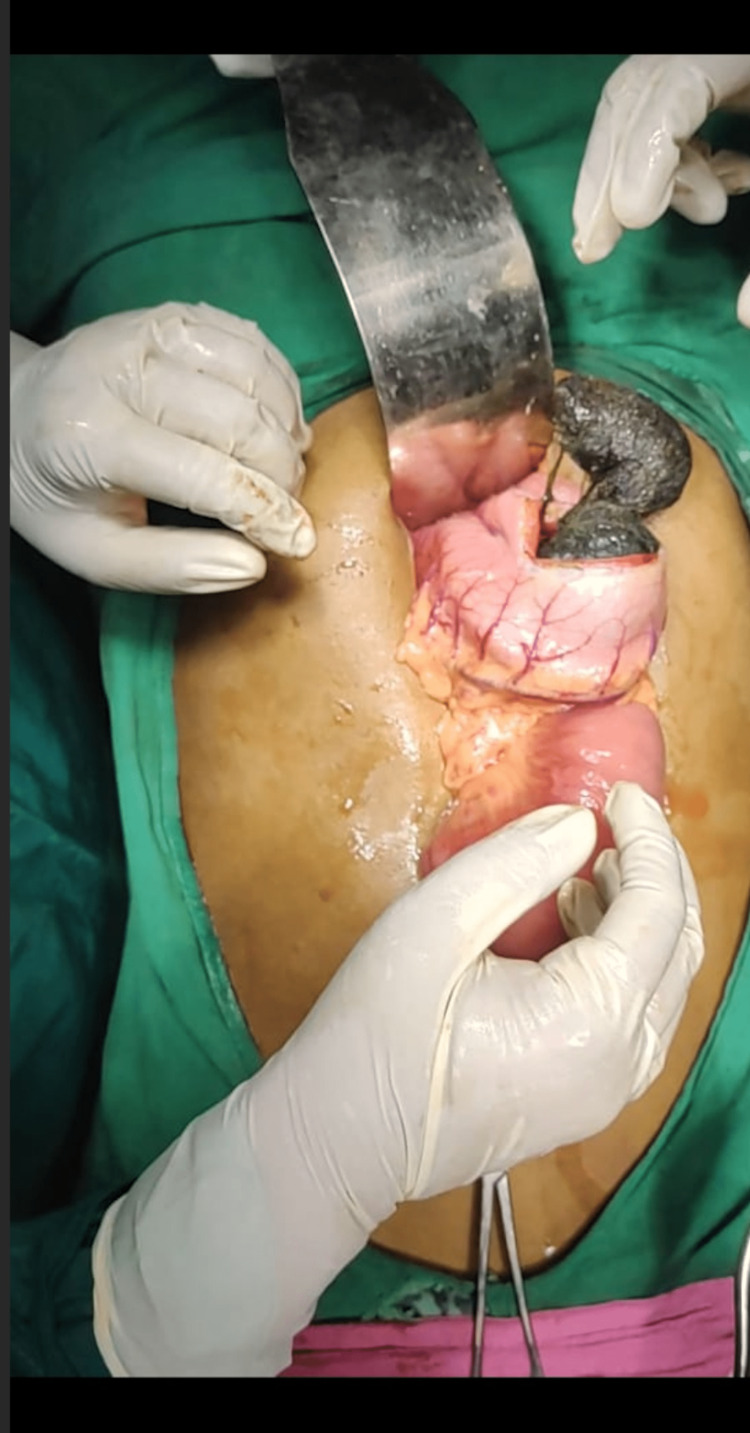
Trichobezoar Removal

**Figure 6:**
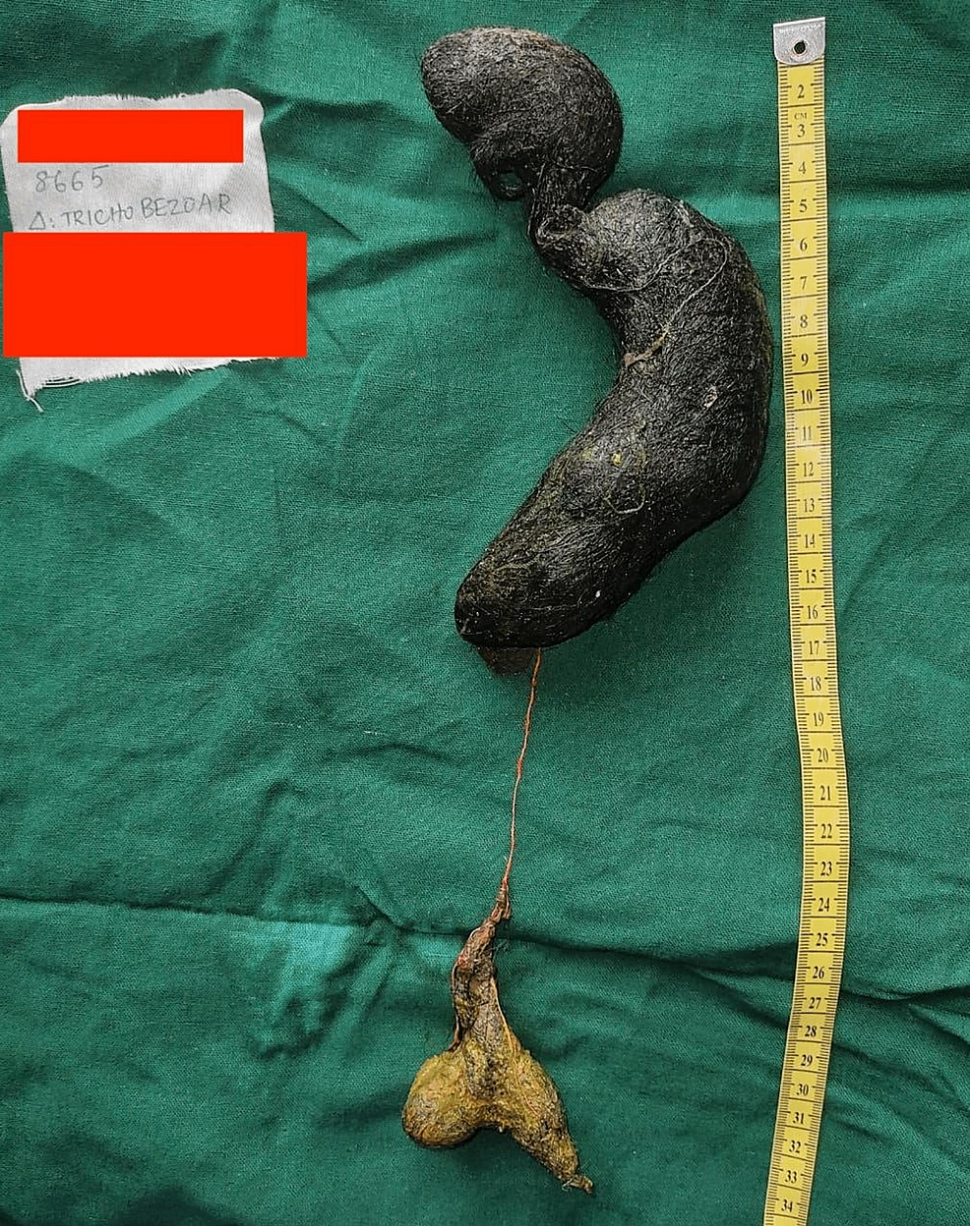
Trichobezoar A stomach-shaped trichobezoar with clumped hair and thread of about 35 cm was identified

Despite best efforts during the endoscopy, only a few hair strands could be removed. An elective laparotomy was performed. During the operation, the stomach, duodenum, and proximal jejunum were found to be dilated. Trichobezoar with clumped hair and thread of about 35 cm was identified which extended from the body of the stomach up to 6 cm beyond the duodenojejunal flexure (Figures 5-6).

Stomach mucosa and proximal jejunal mucosa were found to be inflamed. Unhealthy sloughed-out tissue of about 2×1 cm, indicating mucosal perforation was found at the proximal part of the jejunum at about 7 cm distal to duodenojejunal flexure. This was the point where the trichobezoar ended. Trichobezoar was removed, and primary closure of jejunal perforation with diversion gastrojejunostomy and jejunojejunostomy was done. There were no postoperative complications and the patient was discharged home with outpatient psychiatry follow-up. At the outpatient psychiatry appointment, the patient was started on fluoxetine 10mg daily, then the dose was increased to 20mg daily at the second follow-up. The patient reported improvement in her behavioral symptoms.

## Discussion

Trichobezoar is a condition that is often underdiagnosed and should be considered in the differential diagnosis of chronic abdominal pain and palpable abdominal mass in young female patients, especially if there is a history of behavioral disorders. Trichotillomania is a psychiatric disorder associated with other psychiatric conditions such as kleptomania, pyromania, obsessive-compulsive disorder, skin picking disorder, etc., which are known to be characterized by impaired impulse control [10]. DSM-5 includes trichotillomania as an OCD-related disorder [10]. About 48% of people with trichotillomania orally manipulate their hair [15]. Of them, 33% chew it, and 10% consume the hair in its entirety [15]. Human hair is indigestible due to its slippery surface and enzyme resistance, resulting in its accumulation between the folds of the gastric mucosa [19]. Continued swallowing of hair causes impaction with food, air, and mucus, leading to the development of trichobezoars [19]. About 37.5% of people who engage in trichophagy bear the risk of developing a trichobezoar [15].

In these patients, ultrasound has a relatively low sensitivity in diagnosing gastric bezoars because of the high echogenicity of hair, the presence of trapped air bubbles, and numerous acoustic reflections [19]. According to Lyons et al., with a diagnostic accuracy ranging between 73% to 95%, abdominal CT is the imaging modality of choice for diagnosing a trichobezoar [17]. A well-demarcated, mottled, oval intraluminal mass is visible on CT, and this appearance is caused by the mixture of hair, air, and consumed food. Around 0.3% of gastric bezoars are found incidentally during upper gastrointestinal endoscopy [20]. In our study, CT abdomen showed the bezoar within the distended stomach and endoscopy showed the extent of the bezoar extending beyond the antrum. 

As reported by Gonuguntla et al., endoscopic fragmentation, gastric lavage, enzymatic therapy, or a combination of these methods can be used to extract small trichobezoars [16]. Larger bezoars, on the other hand, can progress to Rapunzel syndrome and require surgical intervention with either laparoscopy and/or laparotomy [19]. In our patient, endoscopic removal was unsuccessful, so we proceeded to laparotomy for trichobezoar removal. Intraoperatively a trichobezoar with clumped hair and thread of about 35 cm extending beyond the duodenojejunal flexure was found. Large bezoars can lead to complications such as perforation of the bowel wall secondary to pressure necrosis [11]. In our case report, the patient was found to have a perforation of the jejunal wall, at the endpoint of the bezoar and it was repaired intraoperatively.

As reported by Memon et al, recurrence in Rapunzel syndrome usually happens when the underlying trichotillomania is not treated [12]. The patient in our case had outpatient psychiatry follow-up and reported improvement in her behavioral symptoms with fluoxetine, there has been no recurrence of this trichobezoar episode to date. To reduce the chances of recurrence, it is critical to treat the underlying psychiatric condition [12]. The characteristics of case reports, which included the symptoms, signs, and the type of intervention performed, are noted in Table ​1.

**Table 1:** Clinical characteristics of case reports of trichobezoar

Author	Year	Patient characteristics	Symptoms and signs	Intervention performed
Lyons et al. [17]	2019	A young female suffering from a psychiatric disorder	Abdominal pain, iron deficiency anemia, failure to thrive, and left upper quadrant mass.	CT - large bezoar. Open laparotomy for removal of bezoar
Sharma et al. [16]	2000	A 14-year-old female with a psychiatric disorder	Loss of hair, scalp pruritis, pain in the abdomen, anemia	A barium meal examination outlined a huge filling defect in the stomach consistent with the diagnosis. Laparotomy and gastrotomy for removal
Folch et al. [18]	2007	Patients with single lung transplantation and recurrence of bezoars	Abdomen distension, bloating, decreased appetite	Endoscopy revealed a large bezoar from the body to the antrum removed by lavage in the first case and fragmentation through endoscopy in the second case.
Gonuguntla et al. [19]	2009	A 5-year-old girl with intellectual disabilities	abdominal pain, vomiting, and a non-tender abdominal mass	A plain radiograph of the abdomen showed multiple air-fluid levels and dilated intestinal loops CT confirmed the presence of bezoar Gastrotomy was performed for the removal
Ventura et al. [11]	2005	5-year-old suffering from child neglect	Cardiorespiratory arrest and declared dead due to sepsis	An autopsy showed an undernourished child, purulent fluid in the abdomen, perforation of the ileum, bezoar in the stomach, and small bowel
Fallon et al. [20]	2013	Case series of females with a mean age of 11.5 years		Multiple imaging modalities and endoscopic evaluation for diagnosis. Exploratory laparotomy as definitive treatment.
Memon et al. [12]	2003	A 12-year-old girl with emotional disorders and recurrence of bezoar	Colicky abdominal pain, lump in the upper abdomen, anemic	An upper GI contrast study showed a large filling defect in the stomach suggestive of gastric bezoar. Removal by gastrotomy.

## Conclusions

Trichotillomania-related trichobezoars should be considered as a differential diagnosis in young patients with a history of psychiatric disorders, who present with abdominal pain. Abdominal CT with contrast is the diagnostic test of choice and if indicated upper gastrointestinal endoscopy should be considered for removal of the bezoar. Surgical intervention may be needed if endoscopy is unsuccessful. It is important to treat the underlying behavioral disorder to prevent a recurrence.
